# MRI features of ovarian metastases from appendiceal tumors and primary ovarian mucinous carcinoma: a comparative study

**DOI:** 10.1007/s11604-026-01992-w

**Published:** 2026-04-16

**Authors:** Masaya Kawaguchi, Hiroki Kato, Tomotaka Kawamura, Takuya Naruse, Tatsuro Furui, Masanori Isobe, Natsuko Suzui, Tatsuhiko Miyazaki, Yoshifumi Noda, Abdelazim Elsayed Elhelaly, Hirohiko Imai, Masayuki Matsuo

**Affiliations:** 1 Department of Radiology, Gifu University, 1-1 Yanagido, Gifu, 501-1194 Japan; 2 Department of Radiology, Ogaki Municipal Hospital, 4-86 Minaminokawacho, Ogaki, 503-0864 Japan; 3 Department of Radiology, Central Japan International Medical Center, 1-1 Kenkonomachi, Minokamo, 505-8510 Japan; 4 Department of Radiology, Gifu Municipal Hospital, 7-1 Kashimacho, Gifu, 500-8513 Japan; 5 Department of Obstetrics and Gynecology, Gifu University, Gifu, Japan; 6 Department of Pathology, Gifu University, Gifu, Japan; 7 Department of Frontier Science for Imaging, Gifu University, Gifu, Japan; 8 Department of Food Hygiene and Control, Faculty of Veterinary Medicine, Suez Canal University, Ismailia, Egypt; 9 Innovation Research Center for Quantum Medicine, Graduate School of Medicine, Gifu University, Gifu, Japan; 10 Center for One Medicine Innovative Translational Research (COMIT), Institute for Advanced Study, Gifu University, Gifu, Japan

**Keywords:** Ovarian metastasis, Appendiceal neoplasms, MRI, Ovarian mucinous carcinoma

## Abstract

**Objective:**

To identify the MRI characteristics of ovarian metastases from appendiceal tumors (OMAT) and compare them with the features of primary ovarian mucinous carcinoma (POMC).

**Materials and Methods:**

This retrospective study included 49 patients with histopathologically confirmed, MRI-detectable, and unruptured OMAT (14 patients with 18 lesions) or POMC (35 patients with 36 lesions) who underwent preoperative MRI. Clinical and MRI findings of the two groups were reviewed and compared.

**Results:**

OMATs were smaller than POMCs (median diameter, 129 vs. 182 mm, *p* < 0.01). Lesion configurations differed between OMATs and POMCs, with the following distributions: purely solid (11% vs. 0%), solid with cystic components (17% vs. 0%), cystic with mural nodules (11% vs. 61%), and purely cystic (61% vs. 39%) (*p* < 0.01). Among cystic lesions, faint septa (94% vs. 31%, *p* < 0.01) were more frequent in OMATs than in POMCs, whereas T1-hyperintense cysts (25% vs. 81%, *p* < 0.01) and a stained-glass appearance (25% vs. 72%, *p* < 0.01) were less frequent in OMATs than in POMCs. Peritoneal dissemination (71% vs. 11%, *p* < 0.01) and abnormal ascites (93% vs. 11%, *p* < 0.01) were also more frequent in OMATs than in POMCs.

**Conclusion:**

OMATs tended to present as smaller, purely cystic lesions with faint septa, whereas POMCs were characterized by larger cystic lesions with T1-hyperintense cysts and a stained-glass appearance.

## Introduction

Ovarian metastasis from malignant tumors is relatively common, accounting for an estimated 15–30% of all ovarian malignancies [1–3]. The most common primary site is the colon, followed by the endometrium, breast, appendix, and stomach [4]. In up to 40% of cases—particularly in patients with colorectal or gastric cancers—the detection of ovarian metastases precedes the diagnosis of the primary tumor [5].

The appendix is a relatively rare primary site, representing 3–7% of ovarian metastases [6]. Appendiceal tumors include mucinous neoplasms, adenocarcinomas, goblet cell adenocarcinomas, and neuroendocrine neoplasms [7]. Ovarian metastases from appendiceal tumors (OMATs) are often difficult to diagnose because of the lack of tumor markers specific to appendiceal neoplasms and characteristic clinical findings [6]. Moreover, distinguishing OMATs from primary ovarian mucinous carcinoma (POMC) is challenging due to histological similarity and overlapping immunohistochemical marker expression [6]. Accurate diagnosis of OMATs is crucial for determining the appropriate extent of surgery, selecting relevant adjuvant chemotherapy, and predicting patient prognosis.

The prognosis for mucinous appendiceal neoplasms differs between low-grade appendiceal mucinous neoplasms (LAMNs) with extra-appendiceal neoplastic epithelium and mucinous adenocarcinomas. The five-year survival rates for LAMN with extra-appendiceal neoplastic epithelium and mucinous adenocarcinoma were 79% and 28%, respectively [8]. Given the prognostic differences among appendiceal tumors, radiographic differentiation is clinically important.

In general, MRI findings of ovarian metastasis are characterized by bilateral involvement with uniform locular size and signal intensity [9–11]. Several studies have reported imaging differences between POMCs and metastatic ovarian carcinomas [9–12], including those originating from gastric and colorectal cancers [13]. Another study showed the ultrasound characteristics of ovarian metastases from LAMN (OMLAMN) [14]. However, to the best of our knowledge, no studies have specifically investigated the MRI findings of OMATs. Therefore, this study aimed to elucidate the MRI characteristics of OMATs and compare them with those of POMCs.

## Materials and methods

### Patients

**Fig. 1:**
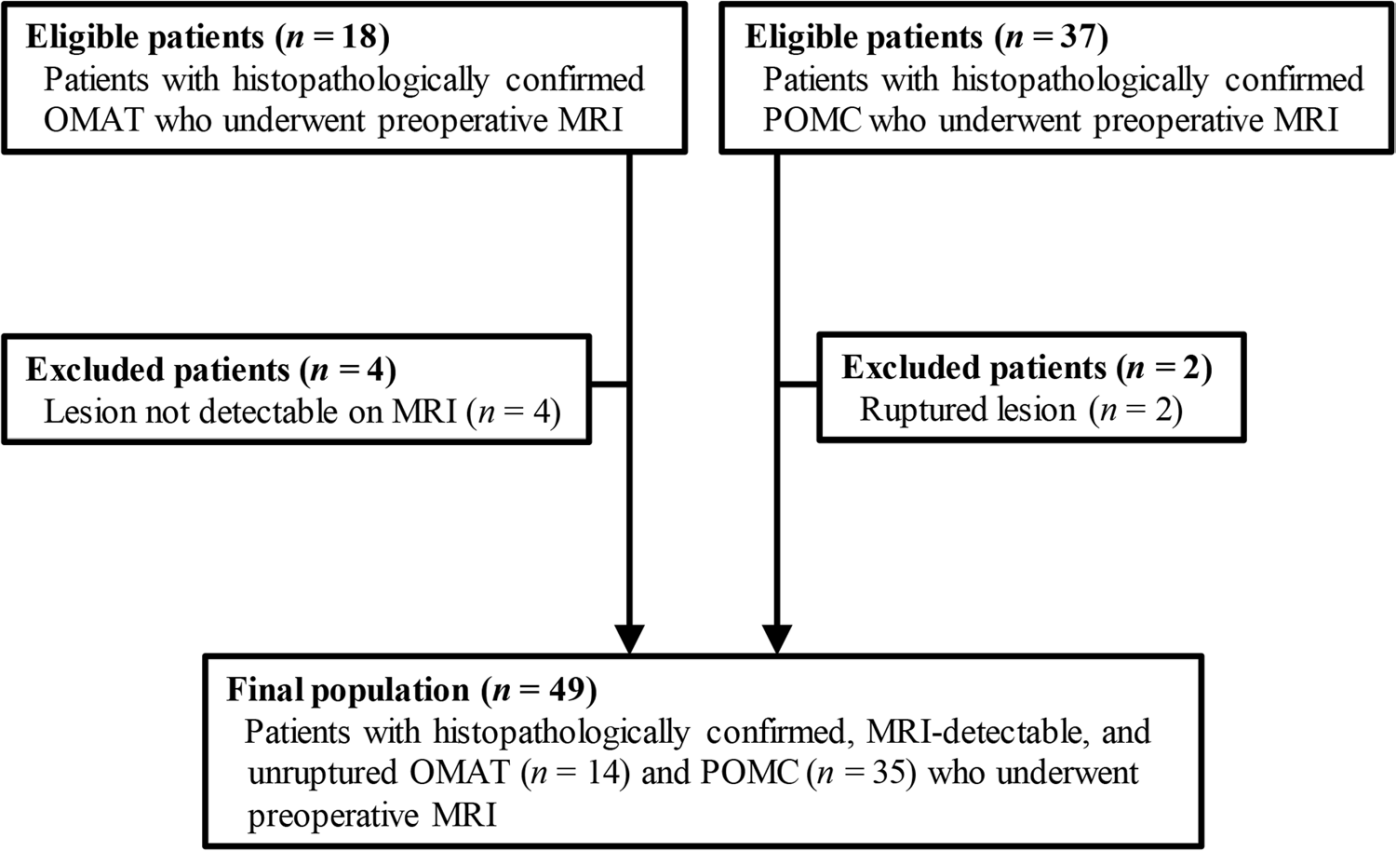
Flow diagram of the study population

This study was approved by the Human Research Committee of our hospital’s institutional review board (2020-093). The requirement for written informed consent was waived by the board because of the retrospective design of the study. The study was conducted in accordance with the Health Insurance Portability and Accountability Act of 1996. Between January 2011 and March 2025, we identified patients who underwent preoperative MRI followed by surgery and had histopathologically confirmed diagnoses. We excluded six patients whose lesions were not detectable on MRI (OMAT: *n* = 4) or were ruptured (POMC: *n* = 2) (Fig. 1). The study included a total of 49 patients: 14 with OMAT (age range, 46–76 years; median age, 65 years) from four Japanese institutions and 35 with POMC (age range, 25–89 years; median age, 65 years) from two Japanese institutions. Among patients with OMAT, histological subtypes included mucinous adenocarcinoma in 7 patients (50%), LAMN in 6 (43%), and non-mucinous adenocarcinoma in 1 (7%).

### MRI Protocols

MRI examinations were performed using either 1.5-T (Intera Achieva 1.5 T Pulsar and Ingenia prodiva 1.5T CS, Philips Healthcare, Best, The Netherlands; Genesis SIGNA and SIGNA Artist, GE Healthcare, USA) or 3.0-T scanners (Intera Achieva 3.0T Quasar Dual and Ingenia 3.0T CX, Philips Healthcare, Best, The Netherlands). Imaging parameters included a section thickness of 4–8 mm with an intersection gap of 1–2 mm and a field of view of 24 × 24–46 × 46 cm. In all 49 patients, acquisitions included axial and sagittal T2-weighted fast spin-echo (TR/TE, 2354–6423/81–147 ms), axial T1-weighted spin-echo (TR/TE, 365–808/8–11 m), and axial fat-suppressed T1-weighted spin-echo (TR/TE, 365–808/8–11 ms) sequences. Axial diffusion-weighted single shot spin-echo echo-planar (TR/TE, 2700–5000/69–80 ms; b-value of 0 and 1000 s/mm^2^) was performed in 44 patients. Axial and coronal or sagittal fat-suppressed gadolinium-enhanced T1-weighted spin-echo (TR/TE, 365–808/8–11 ms) images were obtained in 42 patients after intravenous administration of 0.1 mmol/kg gadopentetate dimeglumine (Magnevist, Bayer HealthCare, Leverkusen, Germany) or gadobutrol (Gadavist, Bayer HealthCare, Leverkusen, Germany). Fat-suppressed dynamic contrast-enhanced T1-weighted images were obtained in 13 patients.

### Imaging analysis

Two radiologists with 25 and 11 years of experience in gynecological imaging, blinded to clinical and pathological data, independently evaluated all MRI images. Any discrepancies were resolved by consensus.

First, the reviewers assessed the presence or absence of peritoneal dissemination, abnormal ascites, and lymphadenopathy. Peritoneal dissemination was defined as nodular or smooth thickening of the peritoneum. Abnormal ascites was defined as ascites extending beyond the uterine fundus and/or filling the pelvic cavity. Lymphadenopathy was defined as a short-axis diameter > 8 mm within the pelvis [15, 16].

Second, the reviewers performed a qualitative assessment that included tumor shape (lobulated or ovoid), predominance (cystic or solid), configuration (purely solid, solid with cystic components, cystic with mural nodules, or purely cystic), and the number of cysts [none (0), unilocular (1), multilocular (2–10 loculi, 11–20 loculi, 21–30 loculi, and ≥ 31 loculi)]. The number of cysts was counted on T2-weighted images. When a cystic component was present, the following cystic features were evaluated: faint septa, faint septa > 50%, thickened septa, mille-feuille sign, fluid-fluid level, flow void, stained-glass appearance, T1-hyperintense cysts, T2-hypointense cysts, and the signal intensity of the largest cyst on T1- and T2-weighted images (low to iso or high relative to the iliopsoas muscle). Faint septa were defined as extremely thin (< 2 mm) septa with high signal intensity relative to the muscle on T2-weighted images. Lesions with faint septa > 50% were defined as those wherein such septa comprised more than half of all septa. The mille-feuille sign was defined as a fine layered structure with layers several millimeters apart and a width/length ratio of ≥ 10/20 mm [12]. Thickened septa were defined as septa thicker than 5 mm. Fluid-fluid level and flow void were evaluated on T2-weighted images [17] and stained-glass appearance was assessed on both T1- and T2-weighted images. T1-hyperintense cysts were considered present when at least one cyst showed hyperintensity relative to the iliopsoas muscle on T1-weighted images [18]. T2-hypointense cysts were considered present when at least one cyst showed hypointensity relative to the iliopsoas muscle on T2-weighted images [17]. The signal intensity of the largest cyst was compared with that of the iliopsoas muscle. When a solid component was present, its configuration (eccentric or centripetal) and hypointensity on T2-weighted images were also evaluated. Eccentric and centripetal configurations were defined as numerous solid components distributed along the inner cystic surface and a small proportion of solid components within the cystic mass, respectively [19].

Finally, one reviewer evaluated the quantitative imaging findings, including the maximum diameter of the entire tumor and solid component, the signal intensity ratios of the largest cyst on T1- and T2-weighted images, the cyst with the highest signal intensity on T1-weighted images, the cyst with the lowest signal intensity on T2-weighted images, and the solid components on T1-, T2-, and contrast-enhanced T1-weighted images. The apparent diffusion coefficient (ADC) value of the solid component was also measured. Regions of interest (ROIs) were placed on cystic and solid components as well as on the iliopsoas muscle to calculate signal intensity ratios. The ratios were obtained by dividing the signal intensity of the cystic or solid component by that of the muscle. ADC values were determined using ADC maps by placing ROIs within the largest solid component, excluding necrotic and cystic areas based on T2- and contrast-enhanced T1-weighted images [16].

### Statistical analysis

All statistical analyses were performed using EZR (Saitama Medical Center, Jichi Medical University, Saitama, Japan) [20]. Quantitative variables of OMATs and POMCs, as well as those of OMLAMNs and other OMATs, were compared using the Mann–Whitney *U* test, whereas qualitative variables were analyzed using Fisher’s exact test. Receiver operating characteristic (ROC) curve analysis was used to evaluate the performance of the imaging findings, and the area under the curve (AUC) was then calculated to determine the optimal cut-off value for distinguishing OMATs from POMCs. A *p*-value of < 0.05 was considered statistically significant. Interobserver agreement for qualitative assessments was evaluated using kappa statistics.

## Results

The clinical and imaging findings of OMAT and POMC patients are summarized in Table 1. Bilateral lesions (29% vs. 3%, *p* < 0.05), peritoneal dissemination (71% vs. 11%, *p* < 0.01), and abnormal ascites (93% vs. 11%, *p* < 0.01) were observed more frequently in OMATs than in POMCs. Left-only lesions were less frequent in OMATs than in POMCs (14% vs. 54%, *p* < 0.05). Regarding tumor markers, CEA levels were higher in OMATs than in POMCs (22 vs. 6.5 ng/ml, *p* < 0.01). No significant differences were observed between OMAT and POMC in terms of age, right-only lesions, CA19-9, CA125, or lymphadenopathy.

**Table 1:** Clinical and imaging findings of OMAT and POMC patients

	OMAT (*n* = 14)	POMC (*n* = 35)	*p* value	Kappa
Age (years)	65 [40–73]	65 [63–72]	0.43	
Laterality				
Bilateral	4 (29)	1 (3)	0.019*	
Left only	2 (14)	19 (54)	0.011*	
Right only	8 (57)	15 (43)	0.365	
Peritoneal dissemination	10 (71)	4 (11)	< 0.001*	0.55
Abnormal ascites	13 (93)	4 (11)	< 0.001*	0.61
Lymphadenopathy	0 (0)	1 (3)	> 0.99	1.00
Tumor marker				
CEA (ng/ml)	22 [14–121] (*n* = 8)	6.5 [1.6–8.6] (*n* = 14)	< 0.001*	
CA19-9 (U/ml)	69 [11–152] (*n* = 9)	100 [24–1127] (*n* = 35)	0.47	
CA125 (U/ml)	157 [44–207] (*n* = 8)	64 [30–211] (*n* = 35)	0.33	

Quantitative data are expressed as medians with interquartile ranges in square brackets

Qualitative data are expressed as raw numbers with percentages in parentheses

*Significant difference was observed between OMAT and POMC (*p* < 0.05)

**Fig. 2:**
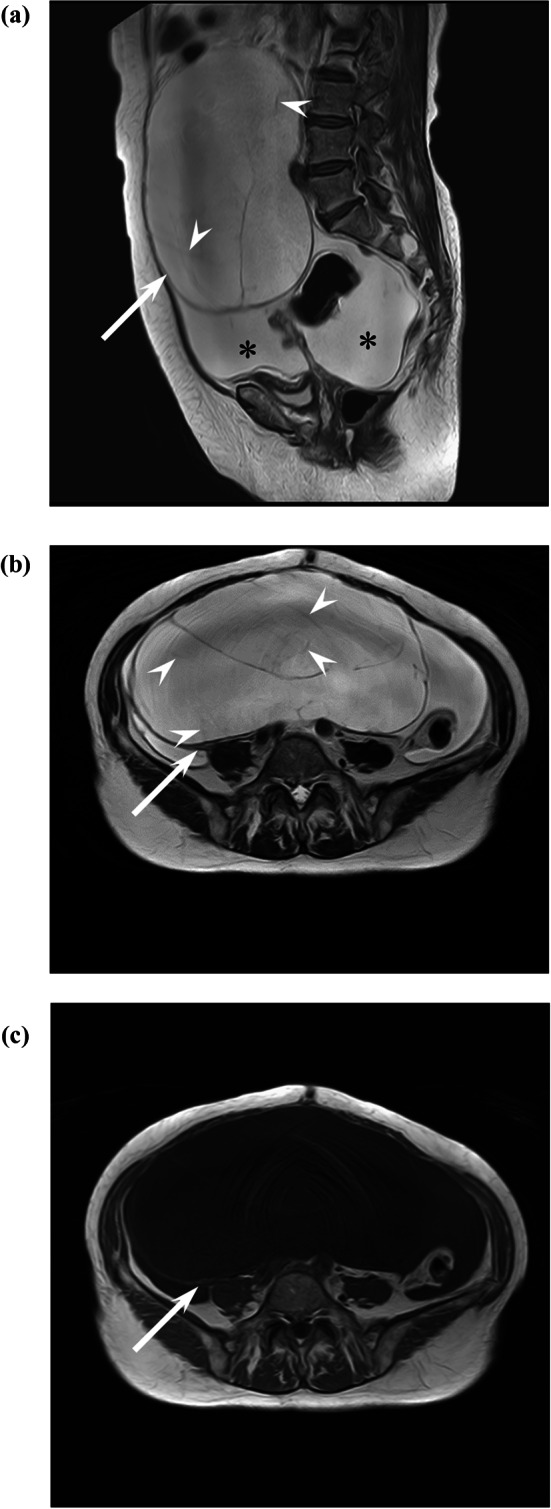
A 71-year-old female with OMLAMN. Sagittal (**a**) and axial (**b**) T2-weighted images showing a multilocular purely cystic mass without solid components (arrow). More than half of the septa appeared faint (arrowhead), and abnormal ascites were present (asterisk). Axial T1-weighted image (**c**) showing a cystic mass (arrow) without a stained-glass appearance

**Fig. 3:**
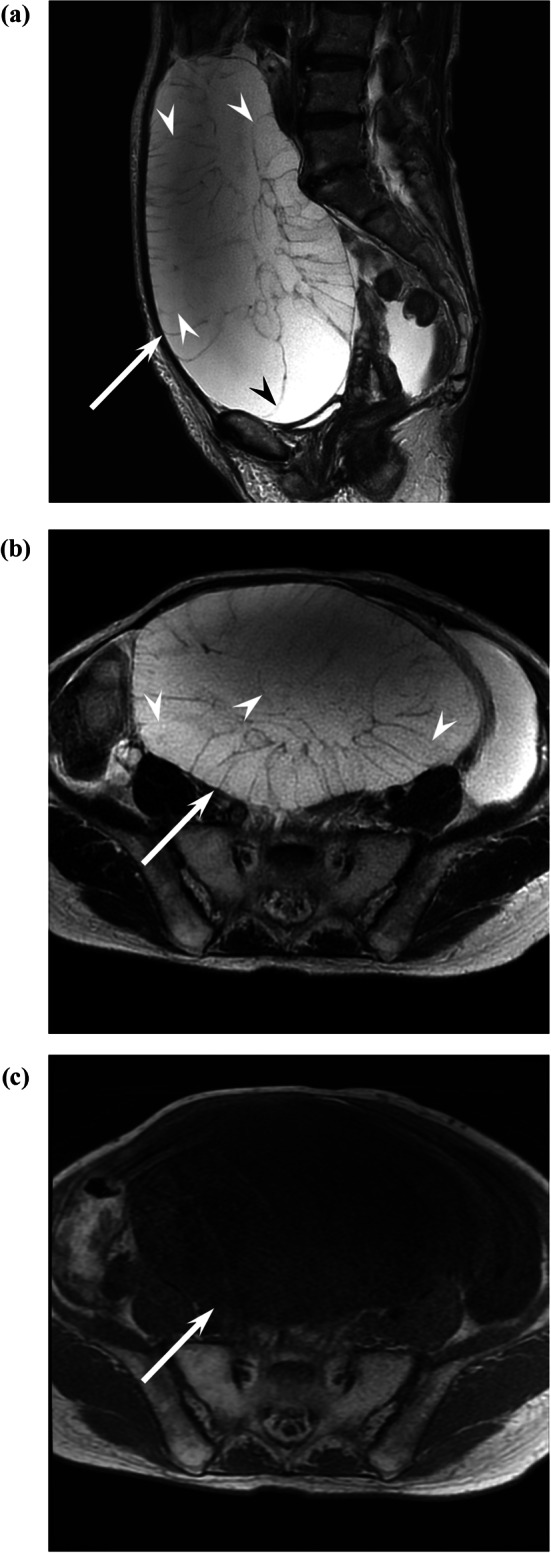
A 64-year-old female with OMLAMN. Sagittal (**a**) and axial (**b**) T2-weighted images showing a multilocular purely cystic mass without solid components (arrow). More than half of the septa were faint (arrowhead). Axial T1-weighted image (**c**) showing a cystic mass (arrow) without a stained-glass appearance

**Fig. 4:**
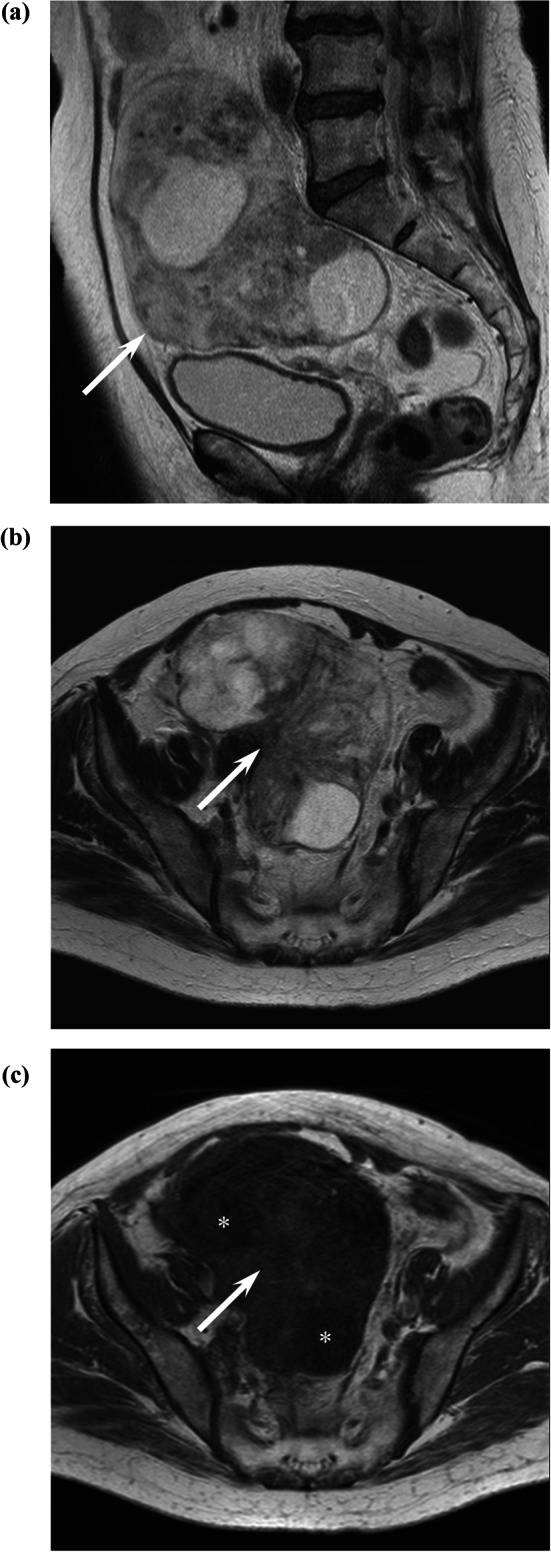
A 65-year-old female with appendiceal mucinous adenocarcinoma. Sagittal (**a**) and axial (**b**) T2-weighted images showing a predominantly solid mass (arrow) with cystic components. Axial T1-weighted image (**c**) showing a solid mass (arrow) with cystic components. The cystic component exhibits low- to iso-signal intensity relative to muscle (asterisk)

**Fig. 5:**
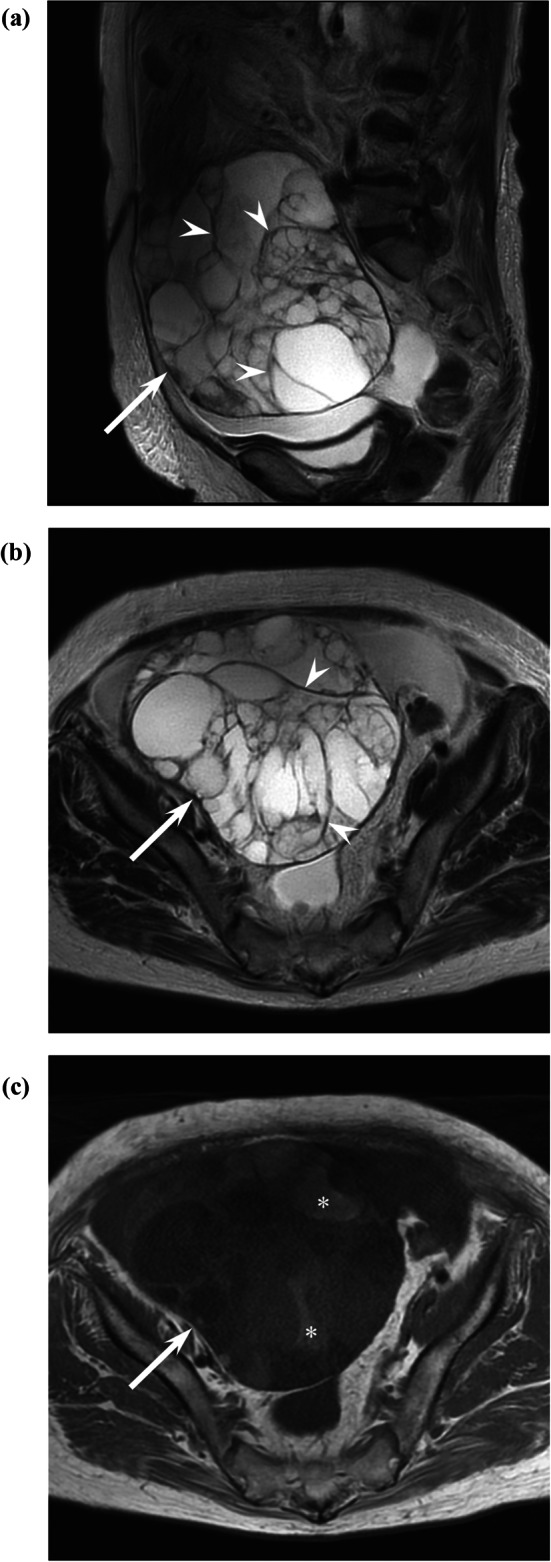
An 82-year-old female with POMC. Sagittal (**a**) and axial (**b**) T2-weighted images showing a multilocular purely cystic mass without solid components (arrow). Neither thickened nor faint septa were observed (arrowhead). Axial T1-weighted image (**c**) showing a cystic mass (arrow) with a stained-glass appearance (asterisk)

Table 2 presents the qualitative MRI findings of OMAT and POMC cases. Bilateral lesions were identified in four patients with OMAT and one patient with POMC, resulting in a total of 18 OMAT cases and 36 POMC cases included in the analysis. A lobulated shape (78% vs. 25%, *p* < 0.01) and predominantly solid lesions (28% vs. 0%, *p* < 0.01) were more frequent in OMATs than in POMCs. Lesion configuration differed between OMATs and POMCs, with the following distributions: purely solid (11% vs. 0%), solid with cystic components (17% vs. 0%), cystic with mural nodules (11% vs. 61%), and purely cystic (61% vs. 39%) (*p* < 0.01) (Figs. 2, 3, 4 and 5). Among cystic lesions, faint septa were more prevalent in OMATs than in POMCs (94% vs. 31%, *p* < 0.01), as were lesions with faint septa > 50% (75% vs. 3%, *p* < 0.01) (Figs. 2 and 3). Significant differences were observed between OMATs and POMCs in the presence of flow voids (44% vs. 11%, *p* < 0.05), stained-glass appearance (25% vs. 72%, *p* < 0.01), and T1-hyperintense cysts (25% vs. 81%, *p* < 0.01). No significant differences were observed in thickened septa, mille-feuille sign, fluid-fluid level, signal intensity of the largest cyst on T1- and T2-weighted images, or T2-hypointense cyst. Among solid lesions, no significant differences were found in configuration or hypointensity on T2-weighted images.

**Table 2:** Qualitative MRI findings of OMAT and POMC cases

	OMAT (*n* = 18)	POMC (*n* = 36)	*p* value	Kappa
Shape–Lobulated	14 (78)	9 (25)	< 0.001*	0.68
Predominance			0.002*	0.64
Cystic	13 (72)	36 (100)		
Solid	5 (28)	0 (0)		
Configuration			< 0.001*	0.69
Purely solid	2 (11)	0 (0)		
Solid with cystic components	3 (17)	0 (0)		
Cystic with mural nodules	2 (11)	22 (61)		
Purely cystic	11 (61)	14 (39)		
Number of cysts			0.001*	0.56
None	2 (11)	0 (0)		
Unilocular	0 (0)	7 (19)		
Multilocular	16 (89)	29 (81)		
*Cystic components*	*n* = 16	*n* = 36		
Number of loculi				
Loculi (> 10)	14 (88)	22 (61)	0.10	0.87
Loculi (> 20)	12 (75)	20 (56)	0.23	0.62
Loculi (> 30)	9 (56)	15 (42)	0.38	0.71
Faint septa present	15 (94)	11 (31)	< 0.001*	0.57
Faint septa (> 50%)	12 (75)	1 (3)	< 0.001*	0.71
Thickened septa	3 (19)	9 (25)	0.73	0.29
Mille-feuille sign	2 (13)	4 (11)	> 0.99	0.40
Fluid-fluid level	0 (0)	6 (17)	0.16	0.74
Flow void	7 (44)	4 (11)	0.030*	0.33
Stained-glass appearance	4 (25)	26 (72)	0.001*	0.73
T1-hyperintense cysts	4 (25)	29 (81)	< 0.001*	0.75
SI of the largest cyst on T1WI			0.11	0.68
Low-to-iso SI	14 (87)	23 (64)		
High SI	2 (13)	13 (36)		
T2-hypointense cysts	1 (6)	10 (28)	0.14	0.41
SI of the largest cyst on T2WI			> 0.99	1.00
High SI	16 (100)	36 (100)		
*Solid components*	*n* = 7	*n* = 22		
Configuration				
Eccentric	1 (14)	13 (59)	0.10	0.35
Centripetal	6 (86)	9 (41)		
Hypointensity on T2WI	0 (0)	3 (14)	> 0.99	0.35

SI = signal intensity, T1WI = T1-weighted image, T2WI = T2-weighted image

Qualitative data are expressed as raw numbers with percentages in parentheses

*Significant difference was observed between OMAT and POMC (*p* < 0.05)

Quantitative MRI findings of OMAT and POMC cases are summarized in Table 3. OMATs were smaller than POMCs (median diameter, 129 mm vs. 182 mm, *p* < 0.01). The signal intensity ratio of the cyst with the highest signal intensity on T1-weighted images was lower in OMATs than in POMCs (0.89 vs. 1.40, *p* < 0.01), whereas that of the solid components on contrast-enhanced T1-weighted images was higher in OMATs than in POMCs (1.81 vs. 1.53, *p* < 0.05). No significant differences were observed in the signal intensity ratio of the largest cyst on T1- and T2-weighted images, the cyst with the lowest signal intensity on T2-weighted images, maximum diameter of solid components, the signal intensity ratio of the solid component on T1- and T2-weighted images, or the ADC value of solid components.

**Table 3:** Quantitative MRI findings of OMAT and POMC cases

	OMAT (*n* = 18)	POMC (*n* = 36)	*p* value
Maximum tumor diameter (mm)	129 [96–151]	182 [158–201]	0.001*
*Cystic components*	*n* = 16	*n* = 36	
SIR of the largest cyst on T1WI	0.82 [0.76–0.92]	0.93 [0.71–1.42]	0.12
SIR of the cyst with the highest SI on T1WI	0.89 [0.79–0.98]	1.40 [1.06–1.81]	0.002*
SIR of the largest cyst on T2WI	5.01 [4.60–7.17]	7.34 [5.46–9.46]	0.14
SIR of the cyst with the lowest SI on T2WI	4.74 [4.33–6.81]	5.43 [4.20–7.44]	0.78
*Solid components*	*n* = 7	*n* = 22	
Maximum diameter of solid component (mm)	91 [60–101]	53 [35–80]	0.11
SIR of the solid components on T1WI	0.98 [0.92–1.04]	1.09 [0.98–1.19]	0.17
SIR of the solid components on T2WI	4.15 [2.77–5.01]	3.37 [2.58–4.37]	0.49
SIR of the solid components on CET1WI	1.81 [1.63–2.34] (*n* = 5)	1.53 [1.26–1.70] (*n* = 22)	0.016*
ADC value of the solid components (×10⁻³ mm²/s)	1.45 [1.26–1.49] (*n* = 5)	1.09 [1.05–1.36] (*n* = 21)	0.052

SIR = signal intensity ratio, T1WI = T1-weighted image, T2WI = T2-weighted image, CET1WI = contrast-enhanced T1-weighted image

Quantitative data are expressed as medians with interquartile ranges in square brackets

*Significant difference was observed between OMAT and POMC (*p* < 0.05)

The results of the ROC curve analysis for differentiating OMAT from POMC are summarized in Table 4. The AUCs for maximum tumor diameter and signal intensity ratio of the cyst with the highest signal intensity on T1-weighted images were 0.77 and 0.79, respectively. The corresponding sensitivity and specificity were 78% and 81% for maximum tumor diameter, and 78% and 85% for the signal intensity ratio, respectively.

**Table 4:** Results of ROC curve analysis for differentiating OMAT from POMC

	AUC (95%CI)	Cutoff value	Sensitivity	Specificity	*p* value
Maximum tumor diameter	0.77 (0.63–0.91)	≤ 152 mm	78	81	< 0.001*
SIR of the cyst with the highest SI on T1WI	0.79 (0.62–0.96)	≤ 1.02	78	85	0.001*

ROC = Receiver operating characteristics, AUC = area under curve, SIR = signal intensity ratio, T1WI = T1-weighted image

Table 5 summarizes the MRI findings of OMLAMN and other OMAT cases. Bilateral lesions were identified in one patient with OMLAMN and three patients with other OMATs, resulting in a total of seven OMLAMN cases and 11 other OMAT cases included in the analysis. All OMLAMNs were purely cystic and solid components were less frequent in OMLAMNs than in other OMATs (0% vs. 64%, *p* < 0.05). Faint septa were observed in all OMLAMNs, whereas stained-glass appearance and T1-hyperintense cysts were not observed (Figs. 2, 3 and 4).

**Table 5:** The MRI findings of OMLAMN and other OMAT cases

	OMLAMN (*n* = 7)	Other OMAT (*n* = 11)	*p* value
Shape–Lobulated	4 (57)	10 (91)	0.25
Predominance			0.10
Cystic	7 (100)	5 (45)	
Solid	0 (0)	6 (55)	
Configuration			0.085
Purely solid	0 (0)	2 (18)	
Solid with cystic components	0 (0)	3 (27)	
Cystic with mural nodules	0 (0)	2 (18)	
Purely cystic	7 (100)	4 (37)	
Solid component	0 (0)	7 (64)	0.013*
Number of cysts			0.50
None	0 (0)	2 (18)	
Unilocular	0 (0)	0 (0)	
Multilocular	7 (100)	9 (82)	
*Cystic components*	*n* = 7	*n* = 9	
Faint septa present	7 (100)	8 (89)	> 0.99
Faint septa (> 50%)	6 (86)	6 (67)	0.59
Thickened septa	1 (14)	2 (22)	> 0.99
Mille-feuille sign	1 (14)	1 (11)	> 0.99
Fluid-fluid level	0 (0)	0 (0)	> 0.99
Flow void	1 (14)	6 (67)	0.15
Stained-glass appearance	0 (0)	4 (44)	0.070
T1-hyperintense cysts	0 (0)	4 (44)	0.070
SI of largest cyst on T1WI			
Low-to-iso SI	7 (100)	7 (78)	0.48
High SI	0 (0)	2 (22)	
T2-hypointense cysts	0 (0)	1 (11)	> 0.99
SI of largest cyst on T2WI			
High SI	7 (100)	9 (100)	> 0.99
Maximum tumor diameter (mm)	152 [108–208]	124 [70–141]	0.12
SIR of the largest cyst on T1WI	0.82 [0.80–0.88]	0.77 [0.73–0.93]	0.84
SIR of the cyst with the highest SI on T1WI	0.83 [0.80–0.89]	0.94 [0.84–1.40]	0.30
SIR of the largest cyst on T2WI	4.89 [4.43–5.79]	6.81 [4.65–8.35]	0.37
SIR of the cyst with the lowest SI on T2WI	4.80 [4.40–5.79]	4.69 [4.33–8.17]	0.84

SI = signal intensity, T1WI = T1-weighted image, T2WI = T2-weighted image, SIR = signal intensity ratio

Qualitative data are expressed as raw numbers with percentages in parentheses

Quantitative data are expressed as medians with interquartile ranges in square brackets

*Significant difference was observed between OMLAMN and other OMAT (*p* < 0.05)

## Discussion

This study compared the MRI findings of OMATs with those of POMCs. Lesion size was smaller in OMATs than in POMCs. Purely cystic configurations were common in OMATs, whereas cystic with mural nodule configurations were more prevalent in POMCs. Among cystic lesions, faint septa were observed more frequently in OMATs than in POMCs, whereas T1-hyperintense cysts and a stained-glass appearance were less frequent in OMATs. Peritoneal dissemination and abnormal ascites were also more frequent in OMATs than in POMCs.

In this study, lesion size was smaller in OMATs than in POMCs. Previous studies have reported that the size of ovarian metastases—more than half of which originated from colon cancer—ranged from 68 to 125 mm [9, 11, 12]. In another study, the median size of OMLAMN was 101 mm [14, 21]. In contrast, the size of POMCs ranged from 138 to 220 mm [9, 11, 17, 22, 23]. Lesion size is therefore a useful parameter for differentiating between OMATs and POMCs.

In the present study, the most common laterality of OMATs was right-only, followed by bilateral and left-only. A previous study reported that the laterality of OMATs was most frequently bilateral (59%), followed by right-only (29%) and left-only (12%) [4]. In contrast, POMCs showed no significant difference between left-only and right-only distribution (45% vs. 46%) and were rarely bilateral (9%) [24]. Thus, OMATs are characterized by bilateral or right-only distribution.

Purely cystic configurations were prevalent in OMLAMN, whereas purely solid and solid with cystic components were observed only in other OMATs. A previous MRI study of ovarian metastases from colon cancer (OMCC) reported that 80% of cases presented as predominantly cystic lesions [13]. Another CT-based study demonstrated that 90% of OMCCs were cystic or mainly cystic [25]. Furthermore, an ultrasonographic study reported that all OMLAMNs (6/6, 100%) appeared as multilocular cysts with high-density mucoid content and no solid components [14]. In the present study, other OMATs exhibited both cystic and solid predominance without distinct or characteristic imaging features, although OMATs overall showed a tendency toward cystic predominance. Although the primary sites differed between the colon and the appendix, and OMATs exhibited a spectrum of imaging appearances, our findings are consistent with those of previous studies. Taken together, predominantly cystic configuration appears to be a characteristic feature of OMLAMNs.

In the present study, faint septa were almost always observed in OMATs. To our knowledge, no previous studies have evaluated septal thickness using CT or MRI. However, ultrasonographic findings have demonstrated that all OMLAMNs (6/6, 100%) contained extremely thin hyperechoic septa [14], which was consistent with our results. Histopathologically, POMCs exhibit destructive stromal invasion with an associated desmoplastic reaction, whereas OMLAMNs are characterized by the absence of destructive invasion and desmoplastic reaction [26, 27]. The faint septa observed in OMLAMNs are likely attributable to the paucity of fibrous connective tissue.

T1-hyperintense cysts and a stained-glass appearance were less prevalent in OMATs than in POMCs. A previous study comparing the MRI features of POMCs and ovarian metastases showed that the latter tended to have a more uniform cystic signal intensity than POMCs, which corroborated our findings [11]. Histopathologically, metastatic tumors generally proliferate more homogeneously than primary tumors, potentially resulting in more uniform mucus production [11]. Differences in cystic signal intensity therefore represent a useful MRI feature for differentiating OMATs from POMCs.

This study has several limitations. First, the sample size was small. Second, contrast-enhanced MRI and diffusion-weighted imaging were not performed in seven and five patients, respectively. Third, only 13 patients underwent dynamic contrast-enhanced MRI, precluding meaningful evaluation of this technique. Fourth, diffusion-weighted imaging data were acquired from six different MRI scanners, as the cases were collected from four institutions. Finally, because OMATs included both OMLAMNs and other OMATs, cystic imaging findings were primarily attributable to OMLAMN findings, whereas solid imaging findings were mainly attributable to other OMAT. Therefore, given the heterogeneity of imaging features within OMATs, multivariable analysis was not performed.

In conclusion, this study identified distinct imaging differences between OMATs and POMCs. OMATs tend to present as smaller, purely cystic lesions with faint septa, whereas POMCs tend to present as larger cystic lesions with T1-hyperintense cysts and a stained-glass appearance. OMLAMNs are characterized by purely cystic multilocular lesions. These MRI findings are valuable for distinguishing OMATs from POMCs and may facilitate more accurate preoperative diagnoses and improved treatment strategies.

## Data Availability

Data will be made available on reasonable request.
